# New 2-Arylbenzofurans from the Root Bark of *Artocarpus lakoocha*

**DOI:** 10.3390/molecules15096548

**Published:** 2010-09-17

**Authors:** Boonchoo Sritularak, Kullasap Tantrakarnsakul, Kittisak Likhitwitayawuid, Vimolmas Lipipun

**Affiliations:** 1 Department of Pharmacognosy and Pharmaceutical Botany, Faculty of Pharmaceutical Sciences, Chulalongkorn University, Bangkok 10330, Thailand; 2 Department of Biochemistry and Microbiology, Faculty of Pharmaceutical Sciences, Chulalongkorn University, Bangkok 10330, Thailand

**Keywords:** *Artocarpus lakoocha*, Moraceae, 2-arylbenzofuran, anti-herpetic activity

## Abstract

Three new prenylated 2-arylbenzofurans – artolakoochol, 4-hydroxy-artolakoochol and cycloartolakoochol – have been isolated from the root bark of *Artocarpus lakoocha* Roxb., Their structures were elucidated through analysis of their spectroscopic data, and their antiherpetic potential was evaluated by the plaque reduction assay.

## 1. Introduction

**Figure 1:**
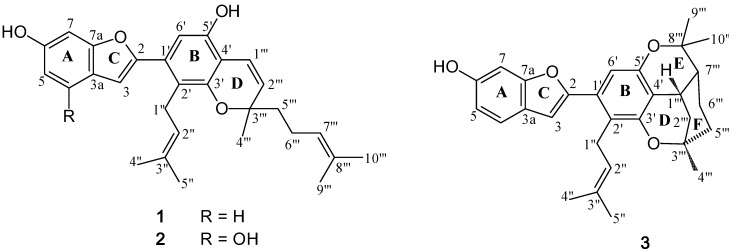
Compounds isolated from the root bark of *Artocarpus lakoocha.*

*Artocarpus lakoocha* Roxb. (Moraceae), locally known in Thai as “Ma-Haad”, is a widely distributed tree in the regions of South and Southeast Asia [1]. Previous phytochemical studies of this plant have revealed the presence of triterpenoids, flavonoids and stilbenes [2,3,4,5], some of which possessed antiherpetic activity [6,7,8]. In an earlier report, we described the isolation of two hitherto unknown 2-arylbenzofuran-type stilbenes from the root of *A. lakoocha* [9]. In this study, a chemical investigation focusing on the root bark was undertaken, and this led to the isolation of three new prenylated 2-arylbenzofurans, namely artolakoochol (**1**), 4-hydroxyartolakoochol (**2**) and cyclo-artolakoochol (**3**) (Figure 1). In addition, these compounds were evaluated for their inhibitory effect against *Herpes simplex* virus using the plaque reduction method.

## 2. Results and Discussion

**Figure 2:**
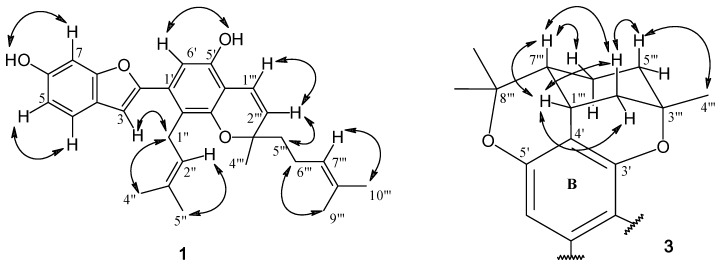
Important NOESY correlations of **1 **and **3**.

**Figure 3:**
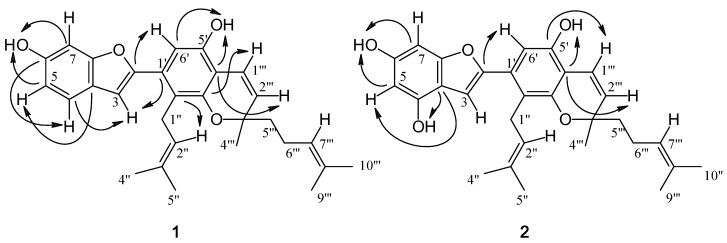
Important HMBC (C→H) correlations of **1 **and **2**.

Compound **1** was isolated as a yellow amorphous solid. The positive HR-ESI-MS exhibited an [M+H]^+^ ion at *m/z* 445.2381 (calcd. for 445.2379; C_29_H_33_O_4_), suggesting the molecular formula C_29_H_32_O_4_. The IR spectrum showed absorption bands for hydroxyl (3,377 cm^−1^), aliphatic (2,966, 2,924 and 2,854 cm^−1^) and aromatic (1,445-1,623 cm^−1^) groups. The UV absorptions at 234 and 339 nm were indicative of a 2-arylbenzofuran skeleton [10,11], and this was supported by the ^1^H-NMR signal at δ 6.72 (1H, d, *J* = 0.5 Hz, H-3), and the ^13^C-NMR signals at δ 105.0 (C-3) and δ 154.4 (C-2) (Table 1) [10]. The ^13^C-NMR and HSQC spectra of **1** displayed 29 carbon signals, corresponding to five methyls, three methylenes, nine methines, and twelve quaternary carbons. The presence of a phenolic group at C-6 on ring A of the 2-arylbenzofuran nucleus was indicated by the OH signal at δ 5.29 (br s, 1H, OH-6, D_2_O exchangable) and the ABM aromatic proton spin system [δ 6.75 (dd, *J* = 8.5, 2.0 Hz, H-5), 6.95 (d, *J* = 2.0 Hz, H-7) and 7.36 (d, *J* = 8.5 Hz, H-4) [9]. This was supported by the NOESY interactions between H-4 and H-5 (Figure 2), and further confirmed by the HMBC correlations from the OH-6 proton to C-5 (δ111.9) and C-7 (δ 98.2) (Table 1 and Figure 3).

**Table 1:** The ^1^H-NMR and ^13^C-NMR data of **1 **(CDCl_3_) and **2** (acetone-*d*_6_).

Position	δ_H_		δ_C_		HMBC (correlation with ^1^H)	
	1	2	1	2	1	2
2	-	-	154.4 (s)	153.2 (s)	6′	3*, 6′
3	6.72 (d, 0.5)	6.86 (d, 1.0)	105.0 (d)	103.1 (d)	4	-
3a	-	-	122.9 (s)	112.3 (s)	3*, 5	3*, 5, 7, OH-4
4	7.36 (d, 8.5)	-	121.2 (d)	152.0 (s)	3	5*
5	6.75 (dd, 8.5, 2.0)	6.29 (d, 2.0)	111.9 (d)	98.6 (d)	7, OH-6	7, OH-6
6	-	-	153.4 (s)	157.6 (s)	4, 7*, OH-6*	5*, 7*, OH-6*
7	6.95 (d, 2.0)	6.49 (d, 2.0)	98.2 (d)	90.4 (d)	5, OH-6	5, OH-6
7a	-	-	155.2 (s)	157.7 (s)	3, 4, 7*	7*, 3
1′	-	-	130.2 (s)	131.9 (s)	3, 6′*	6′*
2′	-	-	120.1 (s)	119.0 (s)	6′, 1′′*, 2′′	6′, 1′′*
3′	-	-	152.3 (s)	153.0 (s)	1′′, 1′′′	1′′, 1′′′
4′	-	-	109.6 (s)	110.2 (s)	6′, 1′′′*, 2′′′, OH-5′	6′, 1′′′*, 2′′′, OH-5′
5′	-	-	149.1 (s)	151.8 (s)	6′*, 1′′′, OH-5′*	6′*, 1′′′, OH-5′*
6′	6.70 (s)	6.80 (s)	106.9 (d)	107.6 (d)	OH-5′	-
1′′	3.45 (d, 6.5)	3.47 (d, 6.5)	25.6 (t)	26.2 (t)	2′′*	2′′*
2′′	5.17 (br t, 6.5)	5.17 (br t, 6.5)	123.6 (d)	124.8 (d)	1′′*, 4′′, 5′′	1′′*, 4′′, 5′′
3′′	-	-	131.2 (s)	131.1 (s)	1′′, 4′′*, 5′′*	1′′, 4′′*, 5′′*
4′′	1.72 (s)	1.74 (s)	18.1 (q)	18.3 (q)	2′′, 5′′	2′′, 5′′
5′′	1.67 (s)	1.65 (s)	25.7 (q)	25.9 (q)	2′′, 4′′	2′′, 4′′
1′′′	6.68 (d, 10.0)	6.74 (d, 10.5 )	117.0 (d)	118.4 (d)	-	-
2′′′	5.57 (d, 10.0 )	5.65 (d, 10.5)	128.6 (d)	128.5 (d)	4′′′, 5′′′	4′′′, 5′′′
3′′′	-	-	78.5 (s)	79.0 (s)	1′′′, 2′′′*, 4′′′*, 5′′′*, 6′′′	1′′′, 2′′′*, 4′′′*, 5′′′*
4′′′	1.36 (s)	1.37 (s)	26.2 (q)	26.6 (q)	2′′′, 5′′′	2′′′
5′′′	1.71 (m)	1.75 (m)	41.3 (t)	42.0 (t)	2′′′, 4′′′, 6′′′*, 7′′′	2′′′, 4′′′, 6′′′*
6′′′	2.10 (m)	2.07 (m)	22.9 (t)	23.6 (t)	5′′′*, 7′′′*	7′′′*
7′′′	5.10 (br t, 7.0 )	5.13 (br t, 7.0 )	124.2 (d)	125.1 (d)	5′′′, 6′′′*, 9′′′, 10′′′	5′′′, 9′′′, 10′′′
8′′′	-	-	131.7 (s)	131.4 (s)	6′′′, 9′′′*, 10′′′*	9′′′*, 10′′′*
9′′′	1.57 (s)	1.57 (s)	17.6 (q)	17.7 (q)	7′′′, 10′′′	7′′′, 10′′′
10′′′	1.65 (s)	1.64 (s)	25.6 (q)	25.8 (q)	7′′′, 9′′′	7′′′, 9′′′
OH-4	-	8.83 (br s)		-		-
OH-6	5.29 (br s)	8.38 (br s)	-	-	-	-
OH-5′	5.19 (br s)	8.48 (br s)	-	-	-	-

*Two-bond coupling.

In ring B one of the *ortho*-position carbon atoms was unsubstituted, as evidenced by the HMBC correlation from C-2 to an aromatic singlet proton at δ 6.70 (H-6′). Further analysis of the ^1^H- and ^13^C- NMR data revealed that a 3,3-dimethylallyl group was present on the other *ortho*-position (C-2′), [^1^Η: δ 3.45 (2H, d, *J* = 6.5 Hz, H_2_-1′′), 5.17 (1H, br t, *J* = 6.5 Hz, H-2′′), 1.67 (3H, s, H_3_-5′′) and 1.72 (3H, s, H_3_-4′′); ^13^C: δ 25.6 (C-1′), 123.6 (C-2′), 131.2 (C-3′), 18.1 (C-4′) and 25.7 (C-5′)]. In support of this, C-2′ (δ 120.1) showed HMBC correlations to H-6′ and H-2′′, and H-3 displayed NOESY interactions with H_2_-1′′. The ^1^H-NMR spectrum of **1** also showed an additional phenolic proton (δ 5.19, 1H, br s, D_2_O exchangeable), which could be assigned to OH-5′ from its 3-bond connectivity to C-6′ and its NOESY correlation to H-6′. Further examination of the remaining ^1^H- and ^13^C-NMR signals suggested that **1** also contained a modified geranyl group that formed a 2-methyl-2-(4-methylpent-3-enyl) chromene structure on ring B [^1^H: δ 1.36 (3H, s, H_3_-4′′′), 1.57 (3H, s, H_3_-9′′′), 1.65 (3H, s, H_3_-10′′′), 1.71 (2H, m, H_2_-5′′′), 2.10 (2H, m, H_2_-6′′′), 5.10 (1H, br t, *J* = 7.0 Hz, H-7′′′), 5.57 (1H, d, *J* = 10.0 Hz, H-2′′′) and 6.68 (1H, d, *J* = 10.0 Hz, H-1′′′); ^13^C: δ 117.0 (C-1′′′), 128.6 (C-2′′′), 78.5 (C-3′′′), 26.2 (C-4′′′), 41.3 (C-5′′′), 22.9 (C-6′′′), 124.2 (C-7′′′), 131.7 (C-8′′′), 17.6 (C-9′′′) and 25.6 (C-10′′′) [12,13]. This unit should be situated at C-3′ and C-4′, and its placement was corroborated by the HMBC correlations from H-1′′′ to C-3′ and C-5′, and from H-2′′′ to C-4′. Based on the above spectral evidence, the structure of **1** was established as shown, and the compound was given the trivial name artolakoochol.

Compound **2**, a white powder, was analyzed for C_29_H_32_O_5_ from its [M+H]^+^ ion at *m/z* 461.2328 (calcd. for 461.2332) in the HR-ESI-MS. Its UV and IR properties were similar to those of **1**, suggesting another 2-arylbenzofuran skeleton. Comparison of the molecular formula of compound **2** with that of **1** showed that **2** should be a hydroxy derivative of **1**. This hydroxyl group should be located at C-4 of ring A, due to the absence of the H-4 resonance and the appearance of signals for H-5 and H-7, each as a doublet (*J* = 2.0 Hz) at δ 6.29 and δ 6.49, respectively in the ^1^H-NMR spectrum. This was confirmed by the HMBC correlations from C-3a (δ 112.3) to OH-4 (δ 8.83, 1H, br s) and H-3 (δ 6.86, 1H, d, *J* = 1.0 Hz) (Table 1).

The ^1^H- and ^13^C-NMR signals for rings B and D of **2** resembled those of **1**. Ring B of **2** was unsubstituted at C-6′, as indicated by the three-bond coupling between H-6′ (δ 6.80, s) and C-2 (δ 153.2) in the HMBC spectrum. The presence of a 3,3-dimethylallyl group at C-2′ of ring B was supported by the HMBC correlations of C-2′ (δ 119.0) with H-6′ and H-1′′ (δ 3.47, 2H, d, *J* = 6.5 Hz). The HMBC correlation between C-4′ (δ 110.2) and H-2′′′ (δ 5.65, 1H, d, *J* = 10.5 Hz) confirmed the attachement of a monoterpene unit at C-4′ that was arranged to form a 2-methyl-2-(4-methylpent-3-enyl) chromene structure. Structure **2** was given the trivial name 4-hydroxyartolakoochol.

**Figure 4:**
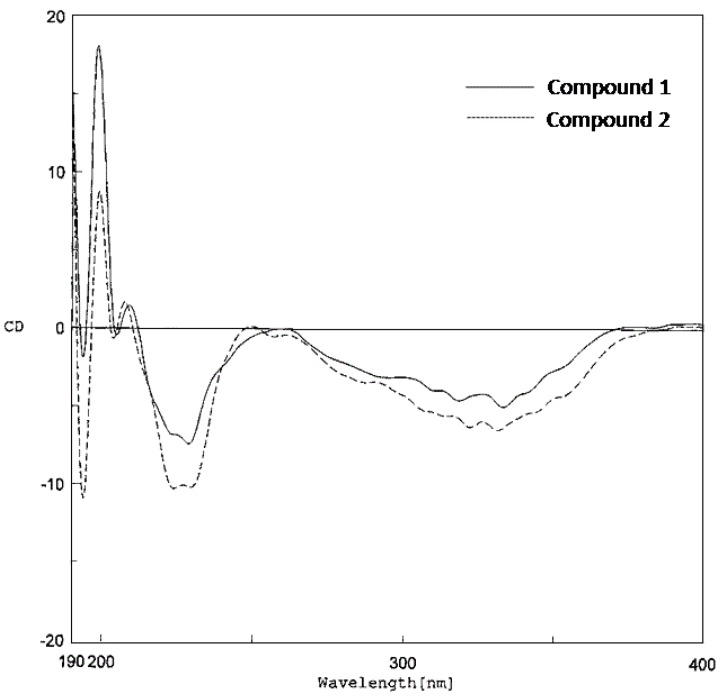
CD data of compounds **1 **and **2**.

Compounds **1** and **2** were optically active with levorotation ([α]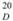 −86.1 and −117.6, respectively). Both shared similar CD properties, displaying a negative Cotton effect at 331 – 334 nm and a negative peak at 227–230 nm (Figure 4), and therefore should have the same stereochemistry at C-3′′′.

Compound **3** was obtained as a yellow amorphous solid. A molecular formula of C_29_H_32_O_4_ was deduced from its [M+H]^+^ ion at *m/z* 445.2391 (calcd. for C_29_H_33_O_4_; 445.2379). The UV absorption and IR bands of **3** were similar to those of **1**, suggesting a 2-arylbenzofuran skeleton. The benzofuran unit (rings A and C) of **3** should have a structure similar to that of **1**, as indicated from the ^1^H-NMR signals of an ABM splitting pattern at δ 6.73 (1H, dd, *J* = 8.0, 2.0 Hz, H-5), δ 6.96 (1H, d, *J* = 2.0 Hz, H-7) and δ 7.36 (1H, d, *J* = 8.0 Hz, H-4), and a doublet signal at δ 6.68 (*J* = 1.0 Hz) assignable to proton H-3 (Table 2). The HMBC correlations of H-4 with C-3 (δ 104.3) and C-6 (δ 153.3) confirmed the presence of a phenolic group at C-6. Similar to **1** and **2**, compound **3** was unsubstituted at position 6′, as evidenced by the ^3^*J* coupling between H-6′ (δ 6.81, 1H, s) and C-2 (δ 155.4).

The ^1^H- and ^13^C-NMR spectrum of **3** also exhibited signals for a 3,3-dimethylallyl group group [^1^H: δ 3.40 (1H, dd,* J* = 14.5, 7.0 Hz, H-1′′_α_), 3.54 (1H, dd, *J* = 14.5, 7.0 Hz, H-1′′_β_), 5.19 (1H, t, *J* = 7.0 Hz, H-2′′), 1.66 (3H, s, H_3_-5′′) and 1.69 (3H, s, H_3_-4′′); ^13^C: δ 25.5 (C-1′′), 123.9 (C-2′′), 130.9 (C-3′′), 18.1 (C-4′′) and 25.8 (C-5′′)] which should be placed at C-2′ due to the HMBC correlations of C-2′ (δ 120.8) with H-1′′. The ^13^C-NMR, HSQC and HMBC spectra of **3** displayed, in addition to the signals for the 2-arylbenzofuran nucleus and the prenyl group, ten carbon signals corresponding to three angular methyls, three methylenes, two methines and two oxygenated quarternary carbons. This indicated that compound **3** also had a monoterpene unit which was attached to C-4′and appeared to form a tricylic structure with the oxygen functionalities on C-3′ and C-5′. The conjugation of a 10-carbon moiety to a di-*ortho* oxygenated aromatic structure to produce a pyran-cyclohexane-pyran system (rings D, E and F) has been recently observed in isorubraine, a monoterpene-chalcone conjugate isolated from the seeds of *Alpinia katsumadai* [14]. Comparison of the ^1^H- and ^13^C-NMR data of **3** with those of isorubraine [14] particularly on the tricyclic partial structure revealed their close similarity. Therefore the monoterpene unit should be connected to ring B by a direct linkage between C-4′ (δ 117.2) and C-1′′′ (δ 28.6) with two ether bridges between C-3′ and C-3′′′, and C-5′ and C-8′′′. This was supported by the HMBC correlation between H_2_-2′′′ (δ 1.82 and 2.21) and C-4′.

**Table 2:** The ^1^H-NMR and ^13^C-NMR data of **3 **(CDCl_3_).

Position	δ_H_	δ_C_	HMBC (correlation with ^1^H)
2	-	155.4 (s)	3*, 6′
3	6.68 (d, 1.0)	104.3 (d)	4
3a	-	123.0 (s)	3*, 5, 7
4	7.36 (d, 8.0)	120.9 (d)	3
5	6.73 (dd, 8.0, 2.0)	111.6 (d)	7
6	-	153.3 (s)	4, 7*
7	6.96 (d, 2.0)	98.2 (d)	5
7a	-	155.5 (s)	3, 4, 7*
1′	-	128.4 (s)	3, 1′′
2′	-	120.8 (s)	6′, 1′′*
3′	-	154.9 (s)	1′′
4′	-	117.2 (s)	6′, 2′′′
5′	-	154.5 (s)	-
6′	6.81 (s)	109.2 (d)	-
1′′ _α_	3.40 (dd, 14.5, 7.0 )	25.5 (t)	2′′*
1′′ _β_	3.54 (dd, 14.5, 7.0 )		
2′′	5.19 (t, 7.0 )	123.9 (d)	1′′*, 4′′, 5′′
3′′	-	130.9 (s)	1′′, 4′′*, 5′′*
4′′	1.69 (s)	18.1 (q)	2′′, 5′′
5′′	1.66 (s)	25.8 (q)	2′′, 4′′
1′′′	2.89 (br t, 2.0)	28.6 (d)	2′′′*, 6′′′
2′′′ax	1.82 (dd, 13.0, 1.5)	35.1 (t)	4′′′
2′′′eq	2.21 (m)		
3′′′	-	74.6 (s)	2′′′*, 4′′′*, 6′′′
4′′′	1.39 (s)	29.2 (q)	-
5′′′ax	1.71 (m)	37.5 (t)	4′′′, 6′′′*
5′′′eq	1.42 (m)		
6′′′ax	0.70 (m)	22.3 (t)	5′′′*
6′′′eq	1.25 (m)		
7′′′	2.05 (m)	46.9 (d)	2′′′, 6′′′*, 9′′′, 10′′′
8′′′	-	83.5 (s)	6′′′, 9′′′*, 10′′′*
9′′′	1.52 (s)	29.8 (q)	10′′′
10′′′	1.04 (s)	23.8 (q)	9′′′
OH-6	4.83 (s)	-	-

*Two-bond coupling.

The arrangement of this monoterpene unit was confirmed by HMBC correlations from C-7′′′ (δ 46.9) to H_2_-2′′′ (δ 1.82, 1H, dd, *J* = 13.0, 1.5 Hz; δ 2.21, 1H, m), H_2_-6′′′ (δ 1.25, 1H, m; δ 0.70, 1H, m), H_3_-9′′′ (δ 1.52, 3H, s) and H_3_-10′′′ (δ 1.04, 3H, s), and from C-3′′′ (δ 74.6) to H_2_-2′′′, H_3_-4′′′ (δ 1.39, 3H, s) and H_2_-6′′′.

The F ring of **3** appeared to have a chair conformation. Its relative configuration and NMR assignments were obtained from detailed analysis of the COSY, NOESY, HSQC and HMBC spectra. At C-2′′′, the double doublet at δ 1.82 (*J* = 13.0, 1.5 Hz) was assigned to the axial proton from its NOESY interaction with H-7′′′, whereas the multiplet at δ 2.21 was assigned to the equatorial, consistent with its long-range (W-type) coupling with equatorial H-5′′′ (δ 1.42, m) observed in the COSY spectrum. The axial proton at C-5′′′(δ 1.71, m), as expected, showed NOESY correlation with H_3_-4′′′. The equatorial proton at C-6′′′ (δ 1.25, m) displayed a NOESY cross peak with H-7′′′. 

**Figure 5:**
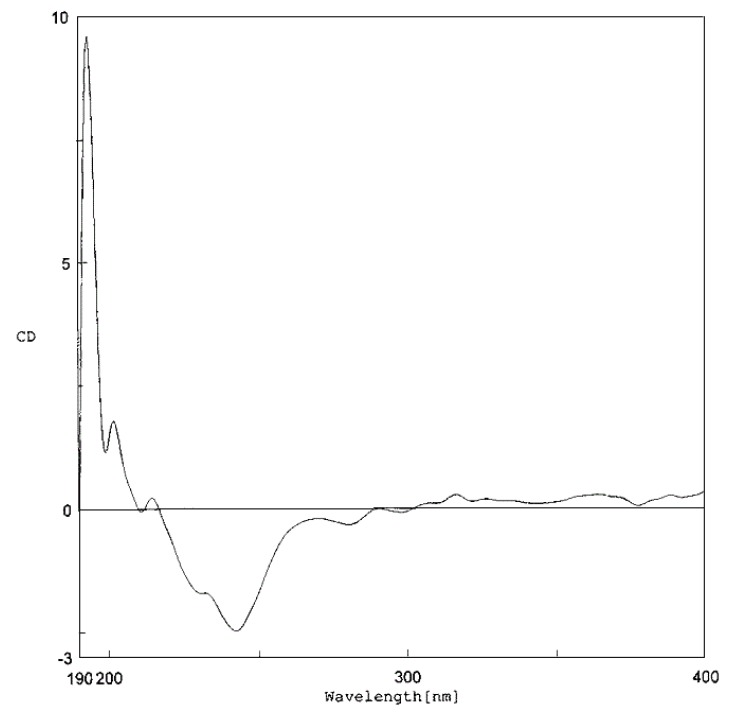
CD data of compound **3**.

Thus, it was concluded that **3** had the structure as shown in Figure 1, and the trivial name cycloartolakoochol was given to the compound. Regarding its optical activity, **3** was found to be dextrorotatory ([α]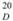 +19.2). In the CD spectrum (Figure 5), it appeared to show a negative Cotton effect at 243 nm, although two small positive peaks at 320 and 370 nm were observed. These findings reflected the influence of the stereochemistry at C-1′′′, which determined the arrangement of the tricyclic (D/E/F) ring system, on the optical properties of **3** as compared with those of **1** and **2**.

Biogenetically, the 2-arylbenzofuran nucleus of **1** and **3** might be derived from 4,3′,5′-trihydroxystilbene (resveratrol), whereas that of **2** could be originated from 2,4,3′,5′-tetrahydroxystilbene (oxyresveratrol) [6,7,8,15]. Compound **3** appears to be a cyclization product of **1**. 

**Figure 6:**
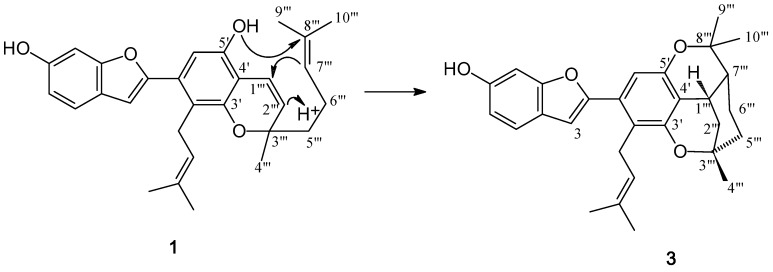
Possible biogenesis of **3** from **1**.

As depicted in Figure 6, this reaction could begin with protonation of C-2′′′, resulting in the formation of a carbocation at C-1′′′. This would be followed by bond formation between C-1′′′ and C-7′′′ to give a carbocation at C-8′′′ that would subsequently undergo nucleophilic attack by OH-5′ to produce rings E and F.

Compounds **1**–**3** were evaluated for their inhibitory activity against *Herpes simplex* virus types 1 and 2 (HSV-1 and HSV-2) using the plaque reduction assay [7,8,9], but they were devoid of activity at the concentration of 100 μg/mL.

## 3. Experimental

### 3.1. General

Optical rotations were measured on a Perkin-Elmer 341 polarimeter, and the CD spectra were recorded on a JASCO J-715 spectropolarimeter. UV spectra were obtained on a Milton Roy Spectronic 3000 Array spectrophotometer, and IR spectra on a Perkin-Elmer FT-IR 1760X spectrophotometer. Mass spectra were recorded on a Micromass LCT mass spectrometer (ESI-TOF-MS). NMR spectra were recorded on a Bruker Avance DPX-300 FT-NMR spectrometer or a Varian Unity INOVA-500 NMR spectrometer. Vacuum-liquid column chromatography (VLC) and column chromatography (CC) were performed on silica gel 60 (Merck, Kieselgel 60, 70-320 mesh), silica gel 60 (Merck, Kieselgel 60, 230-400 mesh) and Sephadex LH-20 (25-100 μm, Pharmacia Fine Chemical Co. Ltd.).

### 3.2. Plant Material

The root bark of *A. lakoocha* Roxb. was collected from the Botanical Garden of Faculty of Pharmaceutical Sciences, Chulalongkorn University, Bangkok, Thailand, in June 2009. Authentication was performed by comparison with herbarium specimens at the Royal Forest Department, Ministry of Agriculture and Co-operatives. A voucher specimen (BS-062552) is on deposit at the Department of Pharmacognosy and Pharmaceutical Botany, Faculty of Pharmaceutical Sciences, Chulalongkorn University.

### 3.3. Extraction and Isolation

Air dried and powdered root bark of *A. lakoocha* (2.4 kg) was successively extracted with EtOAc and MeOH (2 × 15 L, 2 days each) at room temperature, yielding an EtOAc extract (111 g) and a MeOH extract (369 g), respectively. The EtOAc extract was initially subjected to vacuum-liquid chromatography on silica gel (EtOAc-hexane gradient) to give fractions A-M. Fraction E (3.86 g) was separated by CC (silica gel; 15-20% EtOAc-hexane) to give 13 fractions. Fraction 8 (101 mg) was further separated by gel filtration chromatography (Sephadex LH-20, acetone) to give artolakoochol (**1**, 40 mg). Separation of fraction G (3.57 g) was performed on silicagel (10% EtOAc-hexane) and then on Sephadex LH-20 (acetone) to afford cycloartolakoochol (**3**, 6 mg). Fraction K (1.76 g) was separated by CC (silica gel; 30% EtOAc-hexane) to give 18 fractions. Fraction 14 (254 mg) from this column was then subjected to repeated column chromatography over silica gel (MeOH-CH_2_Cl_2_ 1-3%) to give a fraction which was dried and recrystallized from CH_2_Cl_2_ to give 4-hydroxyartolakoochol (**2**, 2.5 mg).

*Artolakoochol* (**1**). Yellow amorphous solid; UV (MeOH): 234 (3.16), 339 (3.23); [α]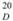 −86.1 (*c* = 0.03, MeOH); CD (MeOH, *c* 0.03): [θ]_193.5_ −3101, [θ]_199_ +30266, [θ]_209_ +2431, [θ]_230_ −12013, [θ]_333.5_ −8337 (Figure 3); IR (film) ν _max_ : 3377, 2966, 2924, 2854, 1623, 1607, 1489, 1445 cm^−1^; HR-ESI-MS: [M+H]^+^ at *m/z* 445.2381 (calcd. for C_29_H_33_O_4_, 445.2379); ^13^C- (125 MHz) and ^1^H-NMR (500 MHz) spectral data see Table 1.

*4-Hydroxyartolakoochol* (**2**). White powder; UV (MeOH): 240 (3.04), 341 (3.02); [α]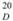 −117.6 (*c* = 0.03, MeOH); CD (MeOH, *c* 0.03): [θ]_193.5_ −14285, [θ]_198.5_ +11842, [θ]_207_ +2242, [θ]_227_ −14326, [θ]_331_ −9227 (Figure 3); IR (film) ν _max _: 3417, 2965, 2920, 2855, 1633, 1609, 1447, 1418 cm^−1^; HR-ESI-MS: [M+H]^+^ at *m/z* 461.2328 (calcd. for C_29_H_33_O_5_, 461.2332); ^13^C- (125 MHz) and ^1^H-NMR (500 MHz) spectral data see Table 1.

*Cycloartolakoochol* (**3**). Yellow amorphous solid; UV (MeOH): 226 (2.50), 316 (2.35); [α]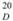 +19.2 (*c* = 0.02, MeOH); CD (MeOH, *c* 0.03): [θ]_194_ +18734, [θ]_199_ +2273, [θ]_202_ +3549, [θ]_214.5_ +447, [θ]_229_ −3300, [θ]_243_ −4939 (Figure 4); IR (film) ν _max_: 3365, 2925, 2873, 2854, 1622, 1489, 1445 cm^−1^; HR-ESI-MS: [M+H]^+^ at *m/z* 445.2391 (calcd. for C_29_H_33_O_4_, 445.2379); ^13^C- (125 MHz) and ^1^H-NMR (500 MHz) spectral data see Table 2.

### 3.4. Assay of Anti-HSV Activity

Antiviral activity against HSV-1 (Strain KOS) and HSV-2 (Strain 186) was determined using the plaque reduction method, as previously described [7,8,9]. Briefly, virus (30 PFU/25 μL) was mixed with complete medium (25 μL) containing various concentrations of test compound and then incubated at 37 °C for 1 h. After incubation, the mixtures were added to Vero cells (6 × 10^5^ cells/well) in 96-well microtiter plates and incubated at 37 °C for 2 h. The overlay medium containing the various concentrations of test compound was added to the Vero cells and incubated at 37 °C in humidified CO_2_ incubator for 2 days. Then, virus growth inhibition was evaluated by counting the virus plaque forming on Vero cells compared with the controls. The cells also were stained with 1% crystal violet in 10% formalin for 1 h. The percent plaque inhibition was determined. Acyclovir was used as positive control.

### 3.5. Cytotoxicity Test

Cytotoxicity was evaluated by incubating Vero cell monolayers with completes medium containing various dilutions of sample for 72 h at 37 °C. Then, cell cytotoxicity was examined by microscopic observation [7,8,9].

## 4. Conclusions

Three new 2-arylbenzofurans: artolakoochol (**1**), 4-hydroxyartolakoochol (**2**) and cyclo-artolakoochol (**3**) were isolated from the root bark of *Artocarpus lakoocha* Roxb. All of the isolated compounds were evaluated for their anti-HSV effect, but they were devoid of activity.
